# Flexible ACEK-Enhanced Capacitive Aptasensor for Rapid Cortisol Detection in Sweat

**DOI:** 10.3390/mi17070800

**Published:** 2026-06-30

**Authors:** Jiuyi Wang, Xiao Lv, Mengjie Yang, Xiaogang Lin, Zhizeng Wang, Jie Jayne Wu

**Affiliations:** 1 Key Laboratory of Optoelectronic Technology and Systems of Ministry of Education of China, Chongqing University, Chongqing 400044, China; 202408021039t@stu.cqu.edu.cn (J.W.); 202308021083t@stu.cqu.edu.cn (X.L.); 202208131095@stu.cqu.edu.cn (M.Y.); 2 Department of Laboratory Medicine, Chongqing Center for Clinical Laboratory, Chongqing Academy of Medical Sciences, Chongqing General Hospital, School of Medicine, Chongqing University, Chongqing 401147, China; 3 Department of Electrical Engineering and Computer Science, The University of Tennessee, Knoxville, TN 37996, USA; jaynewu@utk.edu

**Keywords:** cortisol, alternating current electrokinetic effect, rapid detection, aptamer, capacitive biosensor

## Abstract

Cortisol, as a crucial biomarker reflecting psychological stress and physiological status, requires rapid and sensitive detection for health assessment and disease diagnosis. Conventional methods are time-consuming, operationally complex, and costly, limiting their use for point-of-care testing. This study reports a flexible, aptamer-based capacitive biosensor that exploits alternating current electrokinetics for ultrafast detection of cortisol in small-volume samples. Aptamers are immobilized via Au-S self-assembly on gold interdigitated electrodes on a PET substrate, and ACEK-induced fluid motion and dielectrophoresis rapidly enrich cortisol at the electrode interface, producing measurable interfacial capacitance changes ΔC/C_0_. The experimental results demonstrate that the sensor achieves a detection limit of 0.337 ng/mL in artificial sweat, with a response time within 1 min and a good linear response across the concentration range of 1 to 1000 ng/mL. Requiring only 10 μL of sample, the sensor exhibits good repeatability, specificity, and interference resistance, making it suitable for rapid cortisol level detection. To enhance detection stability, this study designed and integrated a microfluidic chip, enabling efficient sample delivery and stable detection. The system demonstrates strong interference resistance, revealing potential applications in health management and disease monitoring.

## 1. Introduction

Mental health has become a critical determinant of overall well-being in modern society, where increasing life pressures contribute to stress-related disorders [1,2]. Cortisol, the primary stress hormone regulated by the hypothalamic–pituitary–adrenal (HPA) axis, plays a pivotal role in stress response, metabolic regulation, and circadian rhythm maintenance [3]. Clinical studies indicate that abnormal fluctuations in cortisol levels are closely associated with various psychological disorders (such as depression and anxiety) [4,5]. Consequently, cortisol holds significant potential as a vital biomarker for monitoring psychological stress and managing individual health [6,7].

Traditional cortisol detection techniques and methods include enzyme-linked immunosorbent assay (ELISA), radioimmunoassay (RIA), and liquid chromatography-tandem mass spectrometry (LC-MS/MS) [8,9,10], surface plasmon resonance (SPR) [11], and colorimetry [12]. While these methods offer high sensitivity and accuracy, they generally suffer from drawbacks such as complex operation, lengthy detection cycles, high costs, and strong equipment dependency, making them ill-suited for real-time monitoring and point-of-care testing (POCT) under cortisol’s dynamic fluctuations.

With the advancement of nanomaterials [13,14], flexible electronics [15], and micro-fabrication technologies, novel detection techniques such as electrochemical [16,17], optical [18], and transistor-based [19] sensors have emerged, driving cortisol detection toward faster and more miniaturized formats. On this basis, recognition elements, including antibodies [20], aptamers [21,22], and molecularly imprinted polymers [23] have been further introduced, leading to the development of various biosensors [24,25,26,27]. Atul Sharma et al. [28] immobilized antibodies on a vertically layered graphene electrode surface via non-covalent bonds and developed a highly sensitive detection method with a range of 1 fg/mL to 10 ng/mL based on the Fe^3+^/Fe^2+^ redox reaction. Although antibodies are widely used as traditional biomarkers, they still exhibit certain limitations: complex preparation processes, prolonged development cycles, high costs, and susceptibility to cross-reactions during molecular recognition, resulting in insufficient specificity—all of which restrict their application in biosensing. In contrast, aptamers offer significant advantages such as simple synthesis, high stability, and low cost. Ma et al. [21] developed a ratio-based cortisol electrochemical biosensor utilizing a gold electrode modified with multi-walled carbon nanotubes, where aptamers serve as recognition elements combined with methylene blue-labeled zirconium metal–organic frameworks for signal amplification, achieving a detection limit of 0.0046 nM in sweat analysis.

Furthermore, with advancements in microfluidic technology, microfluidic devices have been successfully applied to cortisol detection [29]. Luca et al. [30] reported a paper-based microfluidic device utilizing filter paper to control fluid flow and load reagents for competitive magnetic bead immunoassay analysis of sweat cortisol, achieving a linear detection range of 10–140 ng/mL. Weng et al. [31] developed a portable 3D microfluidic origami biosensor based on a smartphone platform, which successfully detected cortisol concentrations ranging from 10 to 1000 ng/mL in human sweat within 25 min. However, paper-based microfluidic chips fall short in meeting the demands for high-precision quantification, complex sample analysis, and long-term stability, and exhibit prolonged detection times.

To overcome these limitations, rapid detection technologies based on alternating current electrokinetics (ACEK) effect have emerged as an effective strategy for accelerating molecular enrichment at electrode surfaces [32], thereby significantly enhancing sensor response speed and sensitivity [33]. The ACEK effect encompasses three mechanisms: dielectrophoresis (DEP), alternating current electroosmosis (ACEO), and alternating current electrothermal (ACET) effects [34,35,36]. Based on this, this study proposes a rapid cortisol detection method, develops an aptamer electrochemical sensor based on ACEK and microfluidic technology utilizing a flexible fork-finger electrode structure, and establishes a rapid cortisol detection system. Through the optimization of AC excitation voltage, frequency, and aptamer modification concentration, efficient enrichment and highly specific recognition of cortisol were achieved. The sensing interface was characterized by scanning electron microscopy (SEM) and X-ray photoelectron spectroscopy (XPS). Experimental results demonstrated that the sensor exhibited excellent linearity within the concentration range of 1–1000 ng/mL, with a detection limit of 0.337 ng/mL in artificial sweat and a short response time, and only 10 μL of sample was required for detection. This study provides technical support for rapid cortisol detection and offers novel design insights for the development of non-invasive stress monitoring sensors.

## 2. Materials and Methods

### 2.1. Reagents and Equipment

The main reagents used in this study include: 1 × PBS buffer (Solarbio, Beijing, China, purity:analytical reagent (AR) grade), ethanol (Aladdin, Shanghai, China, analytical grade), isopropanol (Macklin, Shanghai, China, with a purity of ≥99.5%), acetone (Kelong, Chengdu, China, purity: AR), nitrogen gas (Shengma Gas, Chongqing, China, with a purity of 99.99%), TCEP solution (Source Leaf, Shanghai, China, with a purity of 99%), 5% bovine serum albumin (BSA) blocking solution (Solarbio, Beijing, China, with a purity of 99%), and 5′-thiol-modified cortisol aptamers (Sangon Biotech, Shanghai, China, High-Performance Liquid Chromatography (HPLC) purified). The experimental instruments used in this study included an ultrasonic cleaner (KQ-300DE, Kunshan Ultrasonic Instruments Co., Ltd., Kunshan, China), a UV-ozone cleaner (SDP-UVT, NovaScan, Inc., Chicago, IL, USA), a vortex mixer (VORTEX 2, IKA-Werke GmbH & Co. KG, Staufen, Germany), an analytical balance (ML204T/02, Mettler-Toledo International Inc., Greifensee, Switzerland), a temperature–humidity chamber (FYL-YS-100L, Beijing Fuyi Electric Appliance Co., Ltd., Beijing, China), micropipettes (Discovery-H series, Shanghai Lichen Bangxi Instrument Technology Co., Ltd., Shanghai, China), and an impedance analyzer (TH2839, Changzhou Tonghui Electronic Co., Ltd., Changzhou, China). Other experimental materials and chemical reagents used in this study were obtained from standard reagent suppliers.

The aptamer was synthesized with a -SH group modified at the 5′ end, and its sequence is: SH-5′-ATG GGC AAT GCG GGG TGG AGA ATG GTT GCC GCA CTT CGG C-3′, which was referenced from the previously published literature [22,37,38].

### 2.2. Pretreatment of Interdigital Electrodes

Polyethylene Terephthalate (PET) was selected as the substrate material for the sensor. The commercial gold interdigital electrode has dimensions of 10 mm × 10 mm, with a line width and spacing of 100 μm × 100 μm, and comprises 10 pairs (20 fingers) of interdigital structures. Prior to sensor fabrication, the interdigital electrodes were subjected to a pretreatment process, including visual inspection, electrode cleaning, UV-ozone surface treatment, and chamber assembly [39].

### 2.3. Preparation of Aptamer Sensor

**Figure 1:**
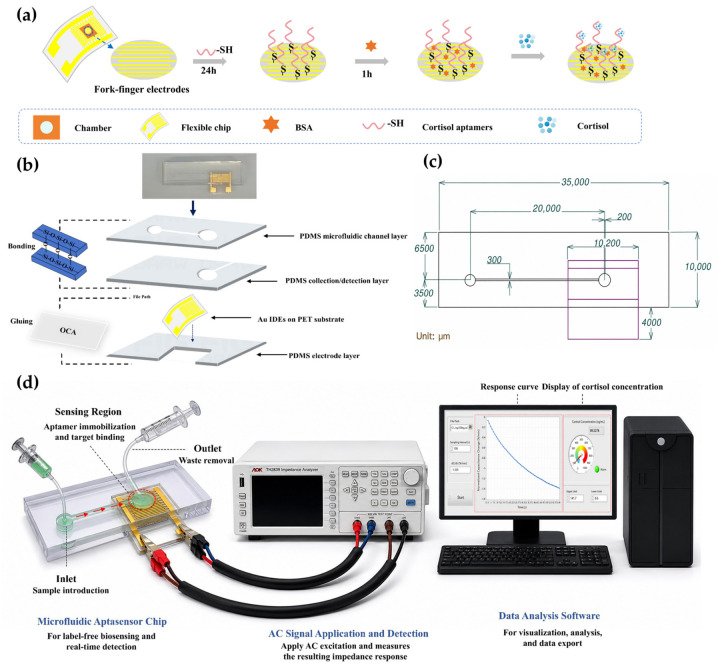
(**a**) Schematic diagram of the aptasensor preparation based on ACEK. (**b**) Schematic and photograph of the microfluidic chip. (**c**) Detailed dimensions of the microfluidic chip. (**d**) Schematic of the rapid cortisol detection system integrating microfluidics, ACEK enrichment, and aptasensor recognition. The red arrows indicate the direction of liquid flow, and the green liquid represents the sample to be tested.

As shown in Figure 1a, the specific fabrication procedure involves the reduction treatment and immobilization of aptamer probes, followed by site blocking with 5% BSA. The resulting functionalized electrodes exhibit a favorable target recognition capability and low non-specific adsorption, enabling their use in subsequent cortisol detection experiments.

### 2.4. Design and Fabrication of Microfluidic Chip

To meet the demand for cortisol detection in sweat samples, a three-layer microfluidic chip was designed and fabricated, as illustrated in Figure 1b. The layers from top to bottom are: the microchannel layer, the collection/detection layer, and the electrode layer. The microchannel layer features an injection port with a diameter of 1 mm and an injection channel measuring 20 mm in length, 300 μm in width, and 170 μm in depth. The collection/detection layer is primarily responsible for solution collection and detection; its detection zone adopts a cylindrical structure with a diameter of 2 mm and a depth of 1 mm, whose edge is tangent to the tip of the underlying finger-like electrode. The sample outlet channel measures 200 μm in width, 170 μm in depth, and 6.5 mm in length. The electrode layer houses a gold-based finger-like electrode cortisol aptamer sensor fabricated on a PET substrate (10.2 mm in length and 6 mm in width), with the groove depth optimized to 170 μm. Detailed dimensions of the chip are shown in the Figure 1c. Schematic of the microfluidic chip fabrication process and bonding process are illustrated in the Figures S1 and S2. The test results of the performance of the prepared microfluidic chip are presented in Figures S3–S6.

### 2.5. Electrochemical Measurement

A rapid detection system for cortisol based on ACEK is shown in Figure 1d. During sample analysis, the system employs a syringe to provide fluid drive force and delivers samples via a medical-grade tube. The measurement adopts a time-scan mode, with a continuous measurement duration of 1 min and a scanning interval of 1 s. To reduce the influence of random experimental errors, each measurement is repeated three times.

## 3. Results and Discussion

### 3.1. Principle of the ACEK Capacitive Aptasensor

The ACEK effect-based electrical biosensor is essentially a capacitive sensor [40], whose detection principle relies on measuring changes in interfacial capacitance within a micro-interdigitated electrode system to characterize the binding process between cortisol and immobilized probes. When micro-interdigitated electrodes are immersed in an electrolyte, a stable electric double layer (EDL) is spontaneously formed at the electrode–electrolyte interface. Specifically, when cortisol molecules specifically bind to the probe on the electrode surface, it induces alterations in the electrical double-layer structure at the electrode–electrolyte interface, thereby resulting in variations in the interfacial capacitance value. To minimize systematic errors and enhance comparability, the relative change in capacitance is commonly used as the detection signal.

### 3.2. Optimization of the Detection Conditions

To maximize the detection performance of the ACEK-based aptamer sensor, the frequency and voltage of the AC electrokinetic signal were optimized through modeling and simulation with COMSOL Multiphysics 6.1, and the aptamer concentration was optimized using the gradient concentration method.

#### 3.2.1. The Voltage of the AC Electrokinetic Signal

As shown in Figure S7, the electric field modulus exhibited a clear positive correlation with the input voltage amplitude. However, a marked increase in the field modulus was observed when the input voltage reached 400 mV. Although theoretically higher voltages could induce a stronger ACEK effect and accelerate fluid flow, an excessively high flow velocity would conversely impair the effective binding between target molecules and the immobilized probes. Based on this consideration, an input voltage amplitude of 400 mV was ultimately selected as the optimal value in this study.

#### 3.2.2. The Frequency of the AC Electrokinetic Signal

As shown in Figure S8, the electric field modulus reached its maximum at a frequency of 15 kHz. At an input frequency of 10 kHz, the electric field modulus was comparable to that at 15 kHz, with only a marginal difference. Based on the equivalent circuit analysis of the gold interdigital electrode–electrolyte system, the impedance variation in the system at low frequencies is predominantly governed by the interfacial capacitance. In addition, lower frequencies offer better immunity to external interference. Taking these factors into consideration, 10 kHz was ultimately selected as the operating frequency for the ACEK effect.

#### 3.2.3. The Aptamer Concentration

As shown in Figure S9, insufficient detection sensitivity was observed at low concentrations (10 μg/mL and 30 μg/mL), while a decrease in sensitivity occurred at the high concentration of 120 μg/mL. In contrast, both 60 μg/mL and 90 μg/mL enabled effective detection across all tested concentrations, with 90 μg/mL yielding the maximum signal response at the high concentration of 1000 ng/mL. After comprehensive consideration of sensitivity and binding efficiency, 90 μg/mL was determined to be the optimal probe concentration.

### 3.3. Sensor Interface Characterization

**Figure 2:**
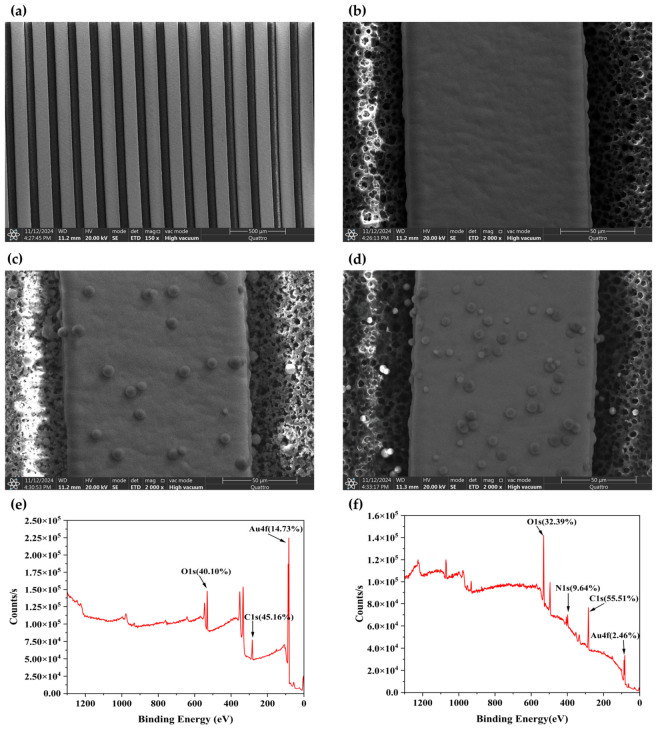
SEM images of electrode surface: (**a**) Bare electrode magnified by 150 times, (**b**) bare electrode magnified by 2000 times, (**c**) electrode modified with probe, (**d**) electrode modified with probe and site-blocked. XPS test energy spectrum graphs: (**e**) Full spectrum graph of the bare electrode after cleaning and (**f**) full spectrum graph of the electrode after probe modification.

To verify the functionalization process of the sensor surface, this study systematically characterized the electrodes at different modification stages using SEM (The instrument used was an environmental scanning electron microscope, manufactured by Thermo Fisher Scientific (West Palm Beach, FL, USA), model Quattro S) and XPS (The instrument used was an X-ray photoelectron spectrometer, manufactured by Thermo Fisher Scientific (West Palm Beach, FL, USA), model Thermo Scientific K-Alpha+). To evaluate the cleaning efficacy of electrode pretreatment, scanning imaging analysis was first performed on the cleaned bare electrodes. Under an acceleration voltage of 20 kV, the electrode surface morphology was observed at magnifications of 150 times (Figure 2a) and 2000 times (Figure 2b). Characterization results demonstrated that the pretreated electrode surfaces were smooth and free from contamination, indicating effective cleaning and providing an optimal environment for sensor fabrication. Under the testing conditions of 20 kV acceleration voltage and 2000× magnification, comparative analyses were conducted between probe-modified electrodes and blocked electrodes. Figure 2c shows granular deposits on the electrode surfaces after aptamer probe incubation, confirming successful fixation of the probes via Au−S bonds over the 24 h incubation period. Figure 2d reveals additional granular deposits of varying sizes on blocked electrodes, verifying the effective deposition of the BSA blocking agent and confirming successful completion of the site-blocking step.

XPS analysis results further confirmed the modification effect: compared with the bare gold electrode (Figure 2e), the aptamer-modified electrode (Figure 2f) surface showed a significant increase in carbon content, the appearance of a characteristic nitrogen signal, and a distinct decrease in the gold signal intensity. This change is highly consistent with the compositional characteristics of aptamer molecules. The pentose sugar structure in nucleotides contributes a large amount of carbon, the nitrogenous bases introduce characteristic nitrogen signals, and the weakened gold signal indicates that the electrode surface has been effectively covered by the aptamer layer. Combined with the elemental analysis results of energy dispersive spectroscopy (EDS), the successful realization of the aptamer modification process on the sensor surface is fully confirmed.

### 3.4. Cortisol Detection in Artificial Sweat

**Figure 3:**
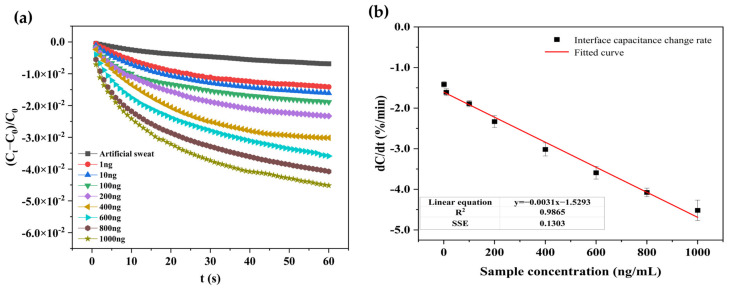
Detection results of cortisol in artificial sweat (*n* = 5): (**a**) The relationship of cortisol samples (Ct − C_0_)/C_0_ at various concentrations over time, and (**b**) the linear fitting diagram of interface capacitance change rate dC/dt and the concentration of cortisol samples.

The recognition performance of the prepared ACEK aptasensor was evaluated to determine its limit of detection (LOD) and detection range for cortisol. To this end, the sensor was exposed to artificial sweat (acidic, mainly composed of sodium chloride, lactic acid, etc.) containing cortisol at concentrations ranging from 1 to 1000 ng/mL, and a quantitative relationship model between cortisol concentration and the detection signal was established. This concentration range covers both the normal physiological fluctuations and abnormal variations of cortisol in sweat, thereby holding potential value for disease diagnosis [16,38,41,42]. The dynamic response curves for different cortisol concentrations are presented in Figure 3a.

As shown in Figure 3b, the rate of interfacial capacitance change (dC/dt) of the system exhibited a good linear relationship with cortisol concentration in the artificial sweat matrix. The linear regression fitting equation was obtained as y=−0.0031x−1.5293, with a coefficient of determination (R^2^) of 0.9865 and a sum of squared errors (SSE) of 0.1303. The LOD in artificial sweat was determined to be 0.337 ng/mL. These metrics confirm that the system possesses satisfactory quantitative detection capability even in a complex physiological matrix.

Table S1 summarizes the comparative parameters, illustrating the performance comparison of representative cortisol sensing methods. The proposed method was compared with other previously reported approaches. It exhibits a relatively low LOD, a short detection time, and a wide linear range.

In this study, the fabricated sensor was employed to detect cortisol in commercially purchased artificial sweat samples to evaluate its practical application performance. The accuracy and precision of cortisol detection in the samples were evaluated by calculating the recovery rate and the relative standard deviation (RSD), respectively. The recovery rate was calculated using the following formula: Recovery (%) = (X_2_ − X_1_)/X_0_ × 100%, where X_2_ is the detected concentration of the spiked sample, X_1_ is the detected concentration of the unspiked sample, and X_0_ is the spiked concentration. The results are presented in Table 1. The detection results showed that the recoveries ranged from 93.595 to 108.394%, while the RSD% values were equal to or lower than 5.626%, indicating that the sensor exhibits high detection accuracy and good reliability in real sample analysis, and thus represents a promising tool for cortisol detection.

### 3.5. Specificity and Repeatability of Sensors

**Figure 4:**
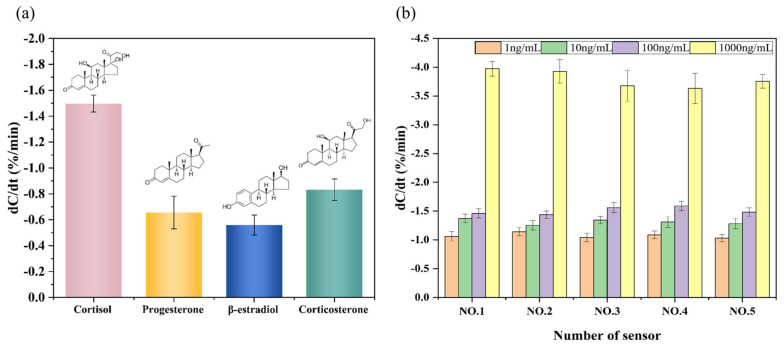
(**a**) Specificity test results of the proposed ACEK-based aptasensor toward cortisol and structurally related analogs. (**b**) Repeatability evaluation of the sensor at different cortisol concentrations (1, 10, 100, and 1000 ng/mL). Error bars represent the standard deviation.

This experiment selected progesterone, β-estradiol, and corticosterone as interferents for comparative analysis. The cortisol concentration was set at 100 ng/mL, while interferent concentrations were uniformly 1000 ng/mL. Figure 4a presents the specific detection results for the four substances. At the high concentration of 1000 ng/mL, the response values for β-estradiol, progesterone, and corticosterone were all below 1%, comparable to the background signal and significantly lower than the response to 100 ng/mL cortisol. Among these, corticosterone exhibited a slightly higher response than the other two interferents due to a structural difference involving only one hydroxyl group compared to cortisol. However, the weak cross-reactivity did not compromise the specificity of the sensor, demonstrating that the designed aptamer sensor possesses excellent specific recognition capability for cortisol.

The relative standard deviation of the sensors across all four concentration gradients was below 5.5%, indicating low dispersion in the test results. This finding confirms excellent consistency among the different sensors under identical detection conditions, thereby enhancing the reliability of the experimental data. To visually demonstrate the reproducibility characteristics, Figure 4b presents the detection results from five sensors at each concentration level. Table S1 summarizes the responses of the sensors in terms of the rate of interfacial capacitance change (dC/dt) to cortisol at four orders of magnitude of concentration.

## 4. Conclusions

This study presented a low-cost, label-free sensor based on the ACEK effect. The sensor employed aptamer-functionalized flexible PET gold interdigital electrodes, which are integrated with a microfluidic chip and data analysis software to form a rapid detection system. The proposed aptasensor displayed good linearity for cortisol detection at concentrations of 1–1000 ng/mL, with LOD as low as 0.337 ng/mL. Furthermore, after adding different concentrations of cortisol to the artificial sweat sample, the recovery rate of the added sample was 93.595–108.394%, with RSDs below 5.626%. These results confirm the accuracy and reliability of the system in complex matrices, suggesting its promising potential for future development as a wearable device for cortisol monitoring.

## Figures and Tables

**Table 1:** The recovery results of cortisol samples in the artificial sweat matrix (*n* = 5).

Sample	Nominal Concentration (ng/mL)	X_1_ (ng/mL)	X_0_ (ng/mL)	X_2_ (ng/mL)	Recoveries (%)	RSD (%)
1	100	110.978 ± 4.157	100	218.373 ± 4.171	108.394	3.369
2	200	215.667 ± 5.808	200	421.444 ± 5.209	103.889	5.626
3	400	396.892 ± 12.380	200	588.083 ± 9.651	93.595	4.708

## Data Availability

Data are contained within the article and Supplementary Materials.

## Supplementary Materials

The following supporting information can be downloaded at https://www.mdpi.com/article/10.3390/mi17070800/s1. Figure S1: (a) Fabrication process of SU-8 silicon wafer mold for microfluidic chip, and (b) the silicon wafer mold of the microfluidic chip; Figure S2: (a) schematic of the microfluidic chip fabrication process, and (b) schematic of the bonding process; Figure S3: diagrams of the solution transport process on the upper surface of the collection/detection layer in the exit channel; Figure S4: diagrams of the solution transport process on the lower surface of the collection/detection layer in the exit channel; Figure S5: Diagrams of the sealing performance test process of the microfluidic chip; Figure S6: diagrams of the solution replacement experiment process on microfluidic chips; Figure S7: electric field magnitude of the model at different voltage amplitudes; Figure S8: electric field magnitude of the model at different frequencies; Figure S9: the response test outcomes of probes with various concentrations in cortisol samples of 1 × PBS; Table S1: experimental results of sensor repeatability (*n* = 5); Table S2: performance comparison of representative cortisol sensing methods.

## Abbreviations

The following abbreviations are used in this manuscript:

HPA	Hypothalamic–pituitary–adrenal
ELISA	Enzyme-linked immunosorbent assay
RIA	Radioimmunoassay
LC-MS/MS	Liquid chromatography-tandem mass spectrometry
SPR	Surface plasmon resonance
POCT	Point-of-care testing
ACEK	Alternating current electrokinetics
DEP	Dielectrophoresis
ACEO	Alternating current electroosmosis
ACET	Alternating current electrothermal
SEM	Scanning electron microscopy
XPS	X-ray photoelectron spectroscopy
BSA	Bovine serum albumin
AR	Analytical Reagent
HPLC	High-Performance Liquid Chromatography
PET	Polyethylene terephthalate
EDS	Energy dispersive spectroscopy
EDL	Electrical double layer
LOD	Limit of detection
RSD	Relative standard deviation
R^2^	Coefficient of determination
SSE	Sum of squared errors
