# Supplementary material for: Flexible ACEK-Enhanced Capacitive Aptasensor for Rapid Cortisol Detection in Sweat
Source: Micromachines (Basel). 2026 Jun 30;17(7):800. doi: 10.3390/mi17070800 (PMC13414359; doi:10.3390/mi17070800)
Supplement: Supplementary file 1 [file micromachines-17-00800-s001.zip › micromachines-4386207-supplementary.pdf]

The Supporting Information for

Flexible ACEK-Enhanced Capacitive Aptasensor for Rapid Cortisol Detection in Sweat

Jiuyi Wang[1], Xiao Lv[1], Mengjie Yang[1], Xiaogang Lin[1]*, Zhizeng Wang[2]* and Jie Jayne Wu[3]

[1] Key Laboratory of Optoelectronic Technology and Systems of Ministry of Education of China, Chongqing University, Chongqing 400044, China; 202408021039t@stu.cqu.edu.cn (J.W.); 202308021083t@stu.cqu.edu.cn (X.L.); 202208131095@stu.cqu.edu.cn (M.Y.)
[2] Department of Laboratory Medicine, Chongqing Center for Clinical Laboratory, Chongqing Academy of Medical Sciences, Chongqing General Hospital, School of Medicine, Chongqing University, Chongqing 401147, China
[3] Department of Electrical Engineering and Computer Science, The University of Tennessee, Knoxville, TN 37996, USA; jaynewu@utk.edu
* Correspondence: xglin@cqu.edu.cn (X.L.); wzhzeng@cqu.edu.cn (Z.W.)

S1. Fabrication process and bonding process of the microfluidic chip

As shown in Fig.S1(a), during the mold and chip fabrication process, SU-8 2100 photoresist is first selected to create a microchannel positive mold on the silicon wafer using conventional soft lithography technology. The following steps include resin homogenization, pre-baking, contact exposure, post-baking, development, and 5 minutes of 150°C film curing; the developed mold surface is treated with trimethylchlorosilane (TMCS) for vapor-phase deposition to prevent adhesion. The successfully fabricated silicon wafer mold is illustrated in Fig.S1(b) and has been stored at 4-21 °C under light protection for subsequent use in manufacturing microfluidic chips.

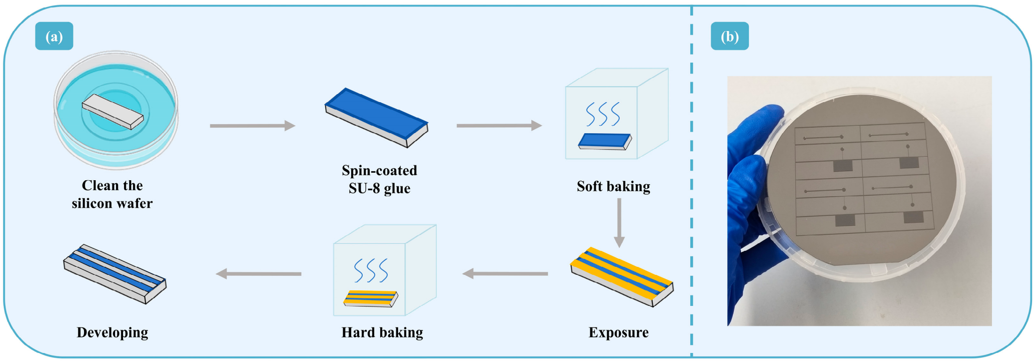

Figure S1.(**a**) Fabrication process of SU-8 silicon wafer mold for microfluidic chip, and (**b**) the silicon wafer mold of the microfluidic chip.

Subsequently, using the prepared SU-8 silicon wafer mold described above, dimethylsiloxane was employed to fabricate the microfluidic chip, as illustrated in Fig.S2 (a). The polydimethylsiloxane (PDMS) prepolymer and curing agent were thoroughly mixed in a mass ratio of 10:1, degassed under vacuum, poured onto the resin mold surface, covered with

a PET film, and flattened, then cured in a 75°C oven for 1.5 hours. After removal and drilling, the microchannel layer and collection/detection layer were treated under oxygen plasma for 40 seconds; the introduction of hydroxyl groups on the surface formed stable Si-O-Si covalent bonds, achieving irreversible bonding between the two layers. A schematic diagram of the bonding process is shown in Fig.S2(b).

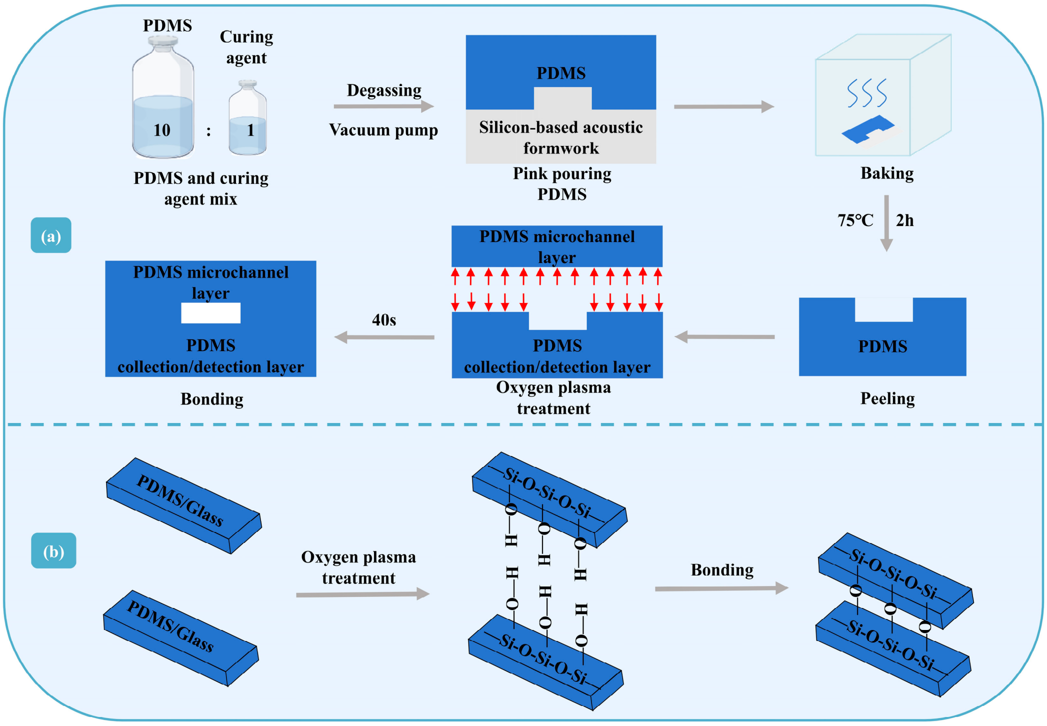

Figure S2. (**a**) Schematic of the microfluidic chip fabrication process, and (**b**) schematic of the bonding process

Finally, due to the raised structure of the electrode's gold layer and the Polyethylene Terephthalate (PET) substrate material, directly bonding the collection/detection layer to the electrode layer requires stringent process specifications and is prone to bonding failure, which compromises chip sealing integrity and may lead to liquid leakage. Therefore, after positioning the aptamer sensor on the electrode layer, a detection aperture is created using an optically clear adhesive (OCA) before bonding the collection/detection layer to the electrode layer. OCA offers advantages such as high transparency, uniform thin-layer adhesion, ease of application, and excellent biocompatibility, making it the ideal choice for bonding these layers. To facilitate detection procedures, the electrode layer is also bonded to the glass slide.

S2. Test results of the performance of the prepared microfluidic chip

S2.1. Selection of the sample outlet channel structure

The sample outlet channel structure selection experiment employed artificial sweat labeled with a red water-based dye compatible with PDMS as the observation solution, which was injected into the microfluidic chip via a syringe, flexible tube, and steel needle. To enable real-time observation of liquid outflow from the sample outlet, none of the validation experiments for the microfluidic chip were connected to a sample recovery device.

The experimental procedure for the sample-outlet structure on the upper surface is shown in Fig.S3. The results demonstrate that this structure offers the following advantages: first, the solution fully fills the detection zone and maintains effective contact with the sensing electrode,

ensuring detection accuracy; second, the solution flows smoothly through the outlet channel without any blockage during transmission, confirming the suitability and reliability of the channel dimensions for sweat samples.

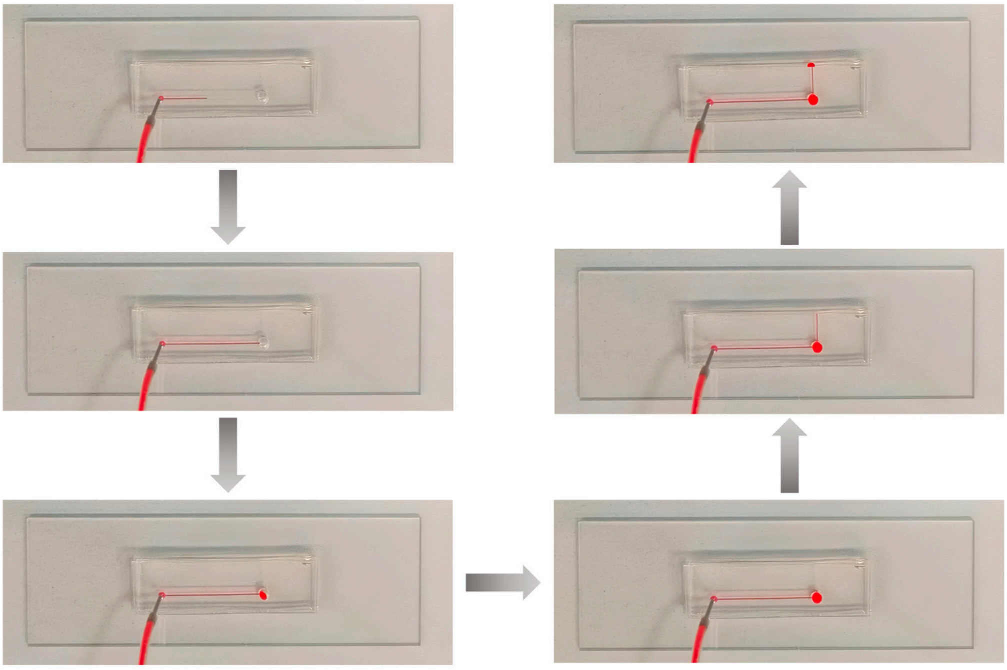

Figure S3. Diagrams of the solution transport process on the upper surface of the collection/detection layer in the exit channel.

Figure.S4 illustrates the experimental procedure and results of the sample collection/detection layer's lower surface structure.

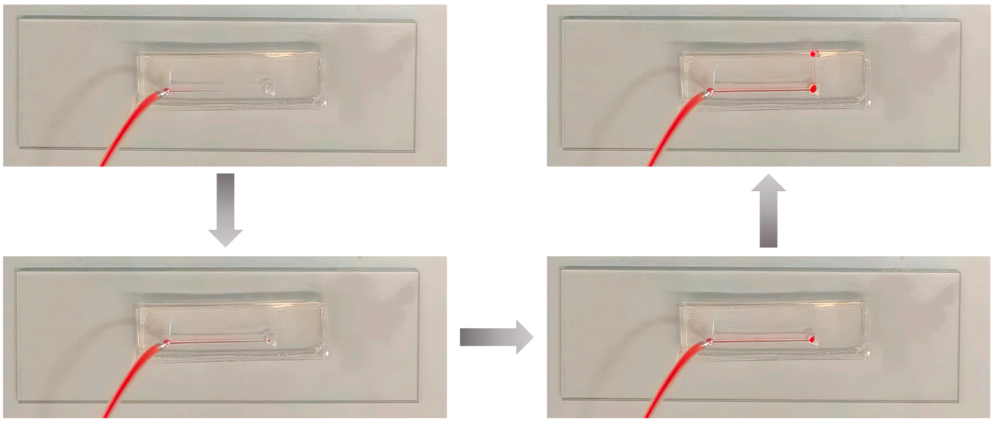

Figure S4. Diagrams of the solution transport process on the lower surface of the collection/detection layer in the exit channel

In contrast, the surface sample exposure structure ensures adequate contact between the solution and the electrodes, thus being designated as the final design for the microfluidic chip. This choice guarantees both detection reliability and fluid controllability.

S2.2. Sealing Performance

The experiment similarly employed artificial sweat containing a red aqueous dye as the observation solution, with sealing performance evaluated through real-time monitoring of fluid flow paths. The sealing test procedure and results are illustrated in Figure.S5: the stained

solution flowed smoothly through the injection channel into the detection zone, completely filled the chamber, and exited via the discharge channel without any leakage. Notably, the solution continued to follow its designed path even as it passed through the electrode protrusion structure, conclusively demonstrating the chip's excellent sealing performance.

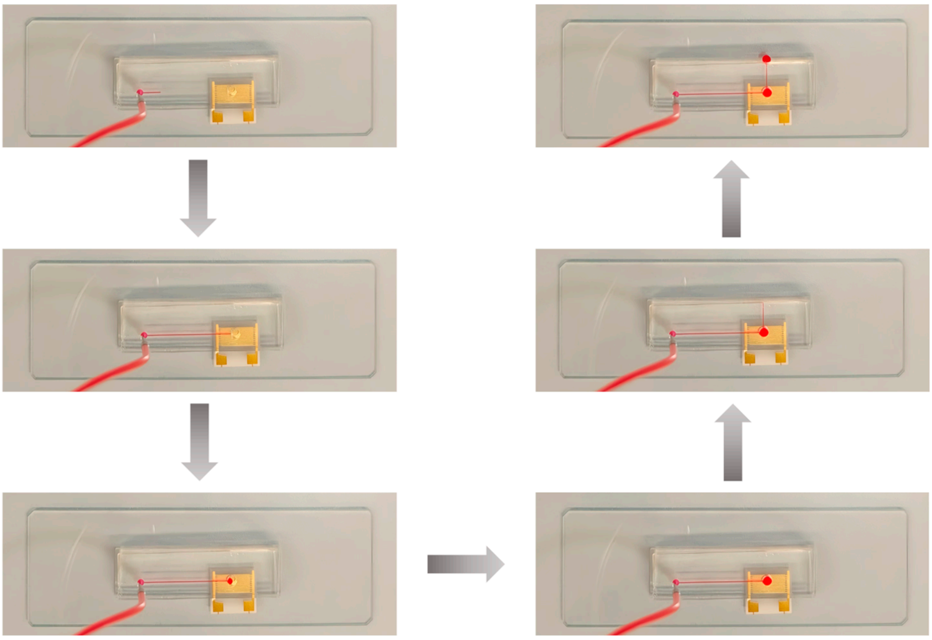

Figure S5. Diagrams of the sealing performance test process of the microfluidic chip

S2.3. Solution displacement

The procedure and results of the solution replacement experiment are shown in Figure.S6. The experiment employed a comparative approach to evaluate the chip's solution renewal performance. First, water-based red dye-labeled artificial sweat completely filled the flow channels, then ultrapure water was injected to simulate the washing process. The results demonstrated that transparent ultrapure water formed a clear fluid interface, achieving effective solution replacement without mixing. This phenomenon confirms that the microfluidic chip exhibits excellent solution renewal capability, meeting the requirements for repeated detection experiments.

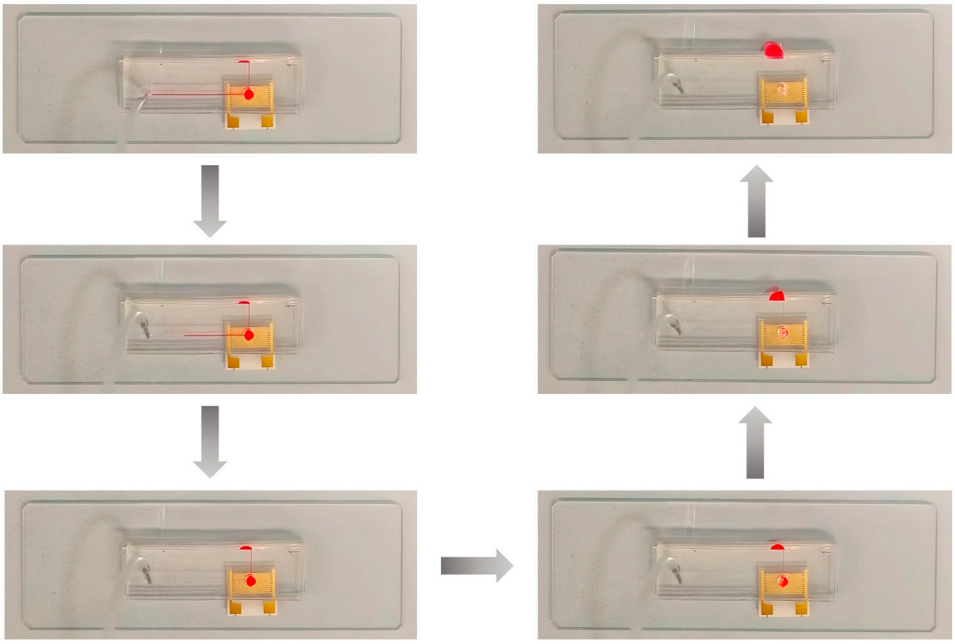

Figure S6. Diagrams of the solution replacement experiment process on microfluidic chips

S3. Simulation results of voltage optimization

In the voltage optimization experiment conducted at a fixed frequency of 10 kHz, sinusoidal AC signals with voltage amplitudes of 1 mV, 10 mV, 100 mV, 400 mV, 700 mV, and 1000 mV were applied sequentially. The electric field modulus at each quarter-cycle was analyzed through simulation, with the results presented in Figure.S7.

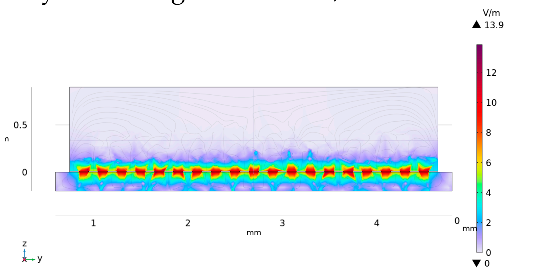

(a)

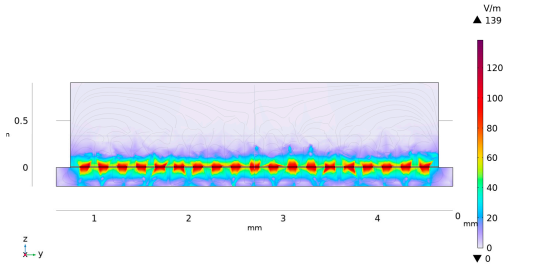

(b)

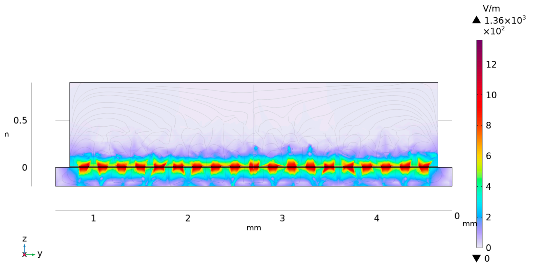

(c)

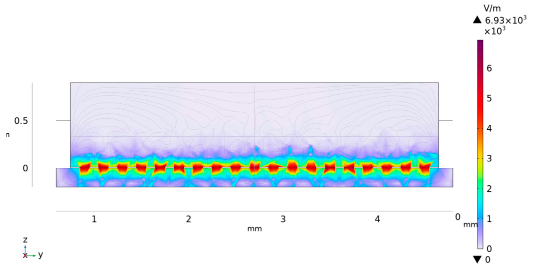

(d)

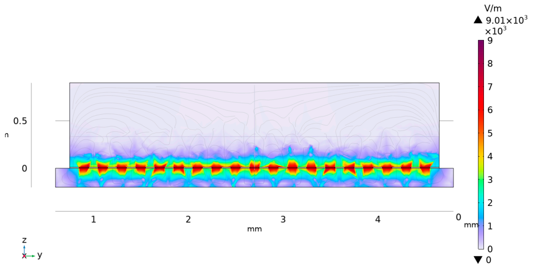

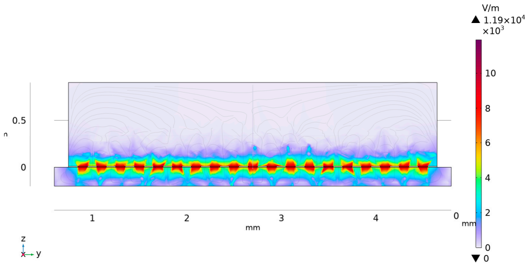

(e) (f)

Figure S7. Electric field magnitude of the model at different voltage amplitudes: (a) 1 mV, (b) 10 mV, (c) 100 mV, (d) 400 mV, (e) 700 mV, (f) 1000 mV

S4. Simulation results of frequency optimization

In the frequency optimization study, simulation analysis was conducted using the model developed during the voltage optimization phase. With a fixed voltage amplitude of 400 mV, sinusoidal AC signals at frequencies of 1 kHz, 5 kHz, 10 kHz, 15 kHz, 20 kHz, 25 kHz, 30 kHz, and 35 kHz were applied, respectively. To ensure consistent experimental conditions, the electric field modulus at one-quarter cycle intervals was selected as the observation metric. The simulation results of electric field mode outputs at different input frequencies are shown in Figure.S8.

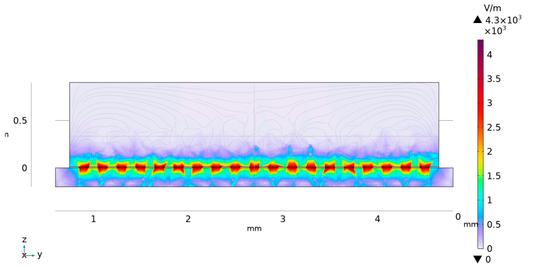

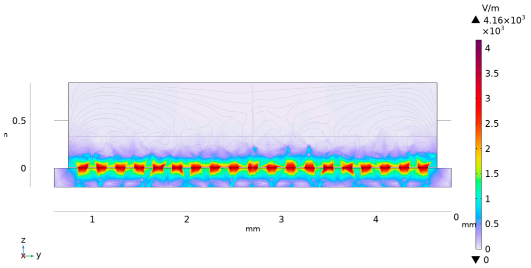

(a) (b)

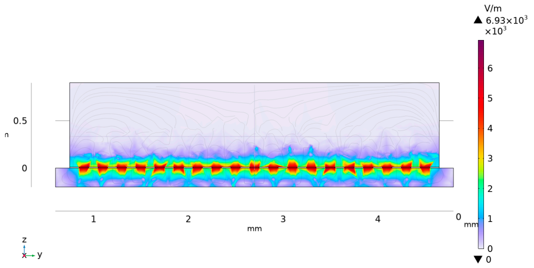

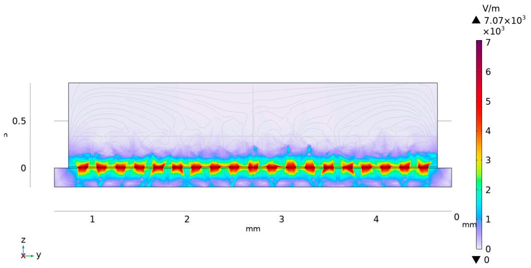

(c) (d)

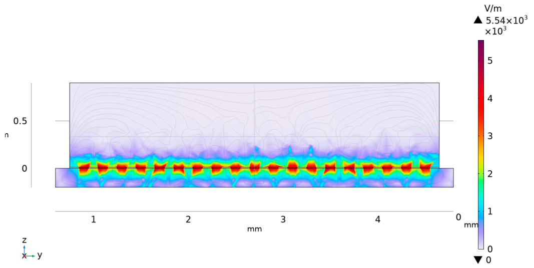

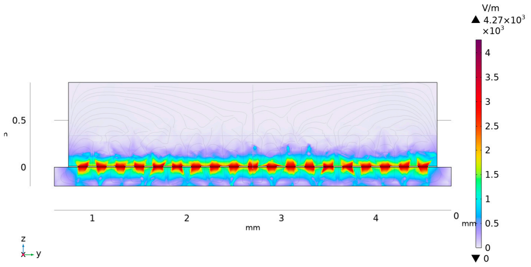

(e) (f)

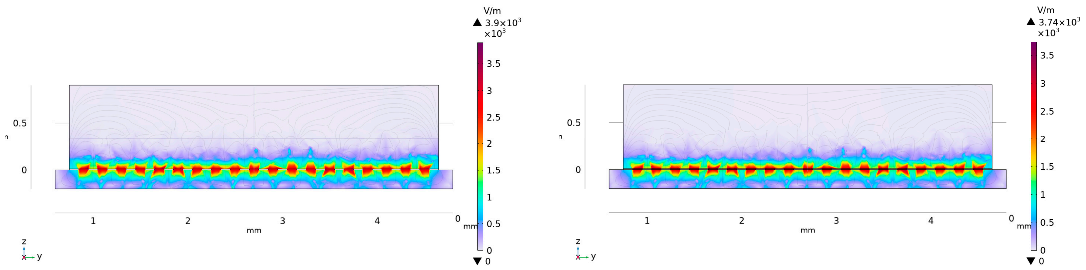

(g) (h)

Figure S8. Electric field magnitude of the model at different frequencies: (a) 1 kHz, (b) 5 kHz, (c) 10 kHz, (d) 15 kHz, (e) 20 kHz, (f) 25 kHz, (g) 30 kHz, (h) 35 kHz

S5. Optimization of the probe concentration

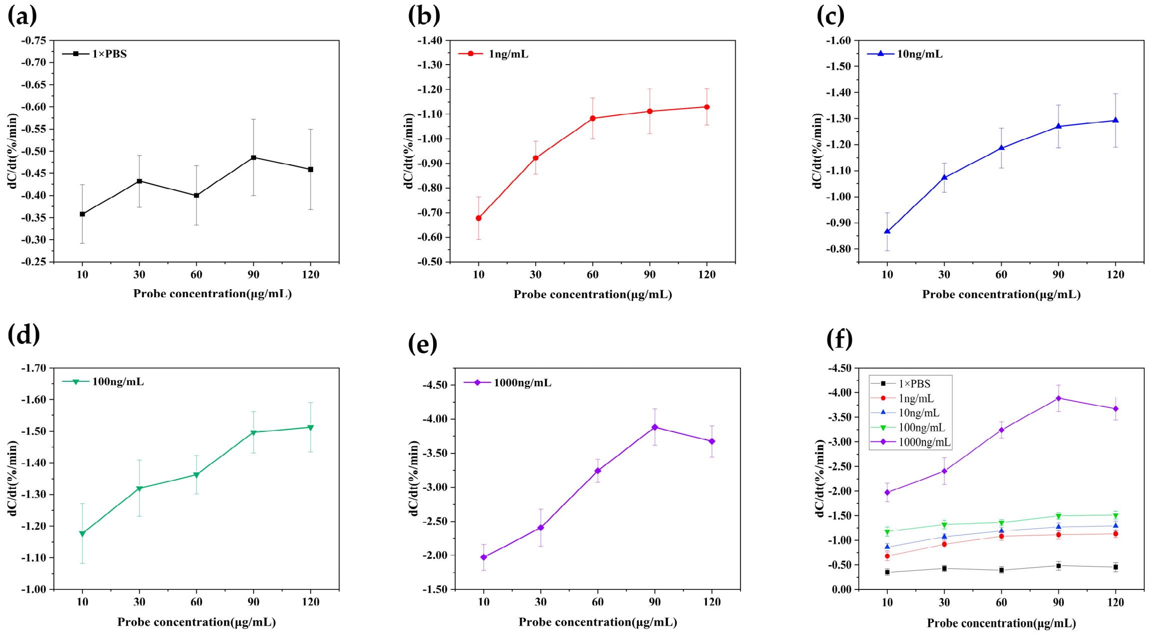

Figure S9. The response test outcomes of probes with various concentrations in cortisol samples of 1×PBS, 1 ng/mL, 10 ng/mL, 100 ng/mL, and 1000 ng/mL (n=5) : (**a**) 1×PBS, (**b**) 1 ng/mL, (**c**) 10 ng/mL, (**d**) 100 ng/mL, (**e**) 1000 ng/mL, (**f**) summary chart. Error bars represent the standard deviation.

S6. Test results for the repeatability of the prepared sensor

The repeatability experiment employed cortisol solutions at four concentration levels: 1 ng/mL, 10 ng/mL, 100 ng/mL, and 1000 ng/mL. Five aptamer sensors were prepared for each concentration. Each sensor performed five parallel measurements on the corresponding concentration samples, and the interface capacitance change rate over 1 minute was calculated. All sensors were fabricated and data processed according to a unified standard to ensure consistent experimental conditions. Table S1 summarizes the interface capacitance change rates of each sensor for the four concentration levels of cortisol.

Table S1. Experimental results of sensor repeatability (n=5)

dC/dt	1	2	3	4	5	Mean Value	Standard Deviation	RSD%

$dC/dt_{1ng/mL}$	-1.059±0.083	-1.142±0.071	-1.044±0.075	-1.088±0.067	-1.031±0.063	-1.073	0.044	4.110
$dC/dt_{10ng/mL}$	-1.371±0.075	-1.254±0.082	-1.343±0.064	-1.310±0.095	-1.281±0.086	-1.313	0.047	3.587
$dC/dt_{100ng/mL}$	-1.460±0.078	-1.438±0.063	-1.562±0.089	-1.590±0.084	-1.480±0.075	-1.506	0.078	5.199
$dC/dt_{1000ng/mL}$	-3.972±0.127	-3.926±0.205	-3.677±0.275	-3.631±0.263	-3.753±0.118	-3.792	0.151	3.982

S7. Performance comparison of representative cortisol sensing methods

Table S2. Performance comparison of representative cortisol sensing methods

Method	Detection range	Limit of detection	Detection Time	Recovery （%）	Ref.
Colorimetry	8–140ng/mL	1ng/mL	-	-	[38]
Electrochemical Immunosensor	5~180 ng/mL	0.54ng/mL	-	94.47~102	[16]
Electrochemistry	2.5~35ng/mL	~1.8ng/mL	-	-	[42]
Microfluidic Fluorescence	10~1000 ng/mL	6.76 ng/mL	25 min	-	[31]
ACEK-Enhanced Capacitive Aptamer	1~1000 ng/mL	0.337 ng/mL	1 min	93.595~108.394	This work
